# Psychosocial Factors Affecting Drug Relapse among Youth in Punjab, Pakistan

**DOI:** 10.3390/jcm12072686

**Published:** 2023-04-04

**Authors:** Najma Iqbal Malik, Sidra Saleem, Irfan Ullah, Syeda Tayyaba Rehan, Domenico De Berardis, Mohsin Atta

**Affiliations:** 1 Department of Psychology, University of Sargodha, Sargodha 40100, Pakistan; 2 Counseling Centre, University of Sargodha, Sargodha 40100, Pakistan; 3 Kabir Medical College, Gandhara University, Peshawar 25120, Pakistan; 4 Dow University of Health Sciences, Karachi 74200, Pakistan; 5 Department of Mental Health, Psychiatric Service of Diagnosis and Treatment, “G. Mazzini” Hospital, Piazza Italia 1, ASL 4, 64100 Teramo, Italy

**Keywords:** conscientiousness, neuroticism, self-efficacy, social support, stigma

## Abstract

The present study was intended to examine the effect of psychosocial factors on the stigma of mental illness among people addicted to drugs who have relapsed in Punjab, Pakistan. A sample composed of 116 people addicted to drugs and who have relapsed was collected through the purposive sampling technique. Certain self-report measures were used to assess the pertinent study variables. The results elucidated that self-efficacy, social support, and conscientiousness were found to be negative predictors and neuroticism a positive predictor of stigma. The results also confirmed social support as being a significant moderator in the relationship between self-efficacy and stigma, and self-efficacy as being a significant moderator in the relationship between social support and stigma. It was safe to conclude that psychosocial factors such as self-efficacy, social support, personality traits and stigma have a significant role in causing addiction relapse. The conclusions made have been discussed thoroughly.

## 1. Introduction

The World Health Organization defines a drug as any material that can alter one or more of its functions when administered to a living organism. Drug misuse is a significant and prevalent global health issue [1]. Drugs decrease an individual’s ability to reason and distort judgment. Several elements are responsible for the attraction of an individual toward alcohol consumption. Amongst these, hereditary elements, and environmental, mental and societal causes are important [2], as well as psychosocial factors such as loneliness, self-efficacy [3], personality traits, social stigma, and neglect from the parents [4]. Additionally, pressure from peers is a significant cause of addiction [5]. Statistics from the study conducted by [6] indicated that nearly 29.5 million adults globally consume unlawful substances, which accounts for about 5.3 per cent of the worldwide population [7]. Drug dependence and addiction treatment include medication and behavioural therapy [8]. However, like with other medical conditions, treatment and rehabilitation are often followed by relapse [9]. Relapse is a characteristic of addiction that is very common, and it is expected that persons trying to overcome dependence may go through one or through numerous relapses before successfully abstaining [10]. Relapse is, however, considered a phase in the cycle-of-change model [11], which presumes that individuals go through a cycle of avoiding, allowing for abstinence, taking active steps to stop, and then reverting. Psychological co-morbidity, liquor use disorder severity, hankering, consumption of other substances, and health and societal elements were constantly found to be significantly correlated with the relapse of alcohol use disorder. On the other hand, helpful social setup elements [12], self-efficacy [13], and elements associated with objective and meaning in life were shielding elements against the relapse of liquor use disorder [14]. Numerous investigations on addicted persons demonstrated the significant influence of mental elements such as self-efficacy, perceived stigma, social support, personality traits, etc., on reducing the hazard of dependence and enhancing its effective intervention. The belief of self-efficacy is one of the mental elements that impacts the success of drug rehab intervention [15]. The belief of self-efficacy is a cognitive-motivational power that defines the individual’s suitable level of coping when their abilities and capabilities are under stress. Lower self-efficacy beliefs deny the person’s issue-solving capability [3].

Furthermore, since drug dependency is an ailment, those affected require socio-psychological support more than those with physiological ailments do. Furthermore, aside from medicinal and medical care, societal and mental support must be a focus for patients with substance misuse, mainly after treatment. The procedure of withdrawing and ending associations with substance-addicted fellows is highly tense, and requires socially supportive relationships and resources [16]. Therefore, support from society is among the elements that have a unique role in sustaining the withdrawal of drug-addicted individuals [17]. Stigma can present an obstacle to life opportunities and personal ambition. It can hinder the ability to find jobs, seek housing [18], obtain indemnity, as well as treatment [19,20]. Personality traits are another critical factor that is responsible for causing a person to use drugs. A study found that neuroticism and conscientiousness personality traits have an impact on causing addiction relapse [21]. Ref [22] stated that a mixture of low conscientiousness and greater neuroticism was related to a greater likelihood of drug relapse, and a combination of a greater level of conscientiousness and a lower level of neuroticism was linked with a lower likelihood of relapse.

Research showed that the accessibility of liquor materials is positive and essential concerning substance use. The dependences have an optimistic and momentous influence on the plight of families [23,24]. Universally spread poverty and fast-increasing joblessness push several individuals into mental strain and anxiety, combined with the scarcity of social networks, which lead them to search for asylum in narcotic medications and substances [25]. Because of a deficiency of access to appropriate healthcare services, persons of lower socio-economic status take inexpensive medicines such as opium for a number of health problems, comprising joint pains, asthma or arthritis, which, after prolonged usage, can lead to drug dependence. Several individuals, particularly youth from financially strong families, become habitual users because of pressure from friends, or an effect sometimes caused by misadventures in search of enjoyment via trying drugs without realising their detrimental influences [26,27]. Something that does not appear on the detector of the related performers and advantages study is a biopsychosocial element, another cause of drug consumption disorders and dependence [28]. The results of another research study recommend that pressure from friends, family constancy and emotional affections are momentous in both exposing individuals to drugs and leading them in the direction of the leaving behaviour. It has been determined that self-motivated drug habit individuals are more likely to be successful in leaving drugs than those admitted into treatment line-ups by force [29]. Denial management counselling helps to reduce denial among individuals with substance abuse disorders [30]. Youth physical and mental health is significant for any nation. If we can understand the significant antecedents of relapse, it will be beneficial to seek or ensure the success of rehab programs aimed at minimising the relapse ratio. Considering the significance of the phenomena, this research is a momentous step in the right direction in that it has tried to find the psychosocial factors responsible for causing addiction relapse. Moreover, this study also finds the moderating role of support from society and self-efficacy in causing addiction relapse.

Following are the hypotheses (H) of the present investigation:

- H1. Self-efficacy and social support will significantly negatively impact stigma among people addicted to drugs who have relapsed.
- H2. There will be a significant negative relationship between conscientiousness and stigma among people addicted to drugs who have relapsed.
- H3. There will be a significant positive relationship between neuroticism and stigma among people addicted to drugs who have relapsed.
- H4. Social support will be a significant moderator in the association between self-efficacy and stigma among people addicted to drugs who have relapsed.
- H5. Self-efficacy will be a significant moderator in the relationship between social support and stigma among people addicted to drugs who have relapsed.

## 2. Methods

### 2.1. Research Design and Sample

A correlational survey research design was deemed appropriate to examine the relationship patterns of phenomena to a greater extent. Males addicted to drugs (*N* = 116) and who have experienced relapse were approached by the researchers, using a purposive sampling technique, from four cities in the Punjab province of Pakistan. Participants were admitted into hospitals, drug addiction centres and rehab centres for their drug rehab treatment related to heroin, alcohol, and opiates. Their age ranged from 16 to 30 years (M = 23.54, SD = 4.68). Data showed that 52.6% of them were married, and 47.4% were unmarried. The onset of drug addiction was found to be at adolescent age (64%), early adulthood (36%) and all were taking drugs for more than two years’ time. Only the in-patient participants from the province of Punjab, Pakistan, who were admitted twice to the Drug Rehab Program due to relapse (on average, the rehab program ranged between thirty and ninety days) were included in the study. Female participants were excluded from the study. 

### 2.2. Instruments

A booklet comprised of a demographic information sheet (name, age, drug addiction type, relapse, information, treatment duration information, etc.), along with self-reported questionnaires including the 10-item General Self-Efficacy Scale [31], 10-item NEO-Five Factors inventory [32], 51-item Social Support Scale [33] and 28-item Stigma Scale [34] were used for data collection. All the scales were self-reported measures anchored on a Likert-type format with sound psychometric properties.

### 2.3. Procedures

Institutional permission to conduct the study was sought from the Department of Psychology, University of Sargodha, Pakistan (SU/PSY/789-S, 22 April 2020), followed by formal permissions from the administrative authorities of the rehab/addiction centres and hospitals. Before collecting data, all the participants were given instructions about the nature and aim of this survey. 

Informed consent was taken from the participants, which confirmed their readiness to partake in the survey. They were also guaranteed that the research would not bring any physical, social, financial, or mental harm and that the data they were going to provide would stay confidential and be used for research purposes only. Demographic information was obtained through the demographic data sheet. Afterwards, a questionnaire dossier, along with verbal as well as written instructions regarding how to respond for the measurement items was provided to them. At the end of the data collection, they were thanked for their contribution. Collected data were screened, and incomplete questionnaires were discarded.

### 2.4. Statistical Analyses

The SPSS-24 version was used to analyse the data. Reliability analysis was executed to tap the psychometric soundness of scales, whereas Pearson product–moment correlation was executed to provide an initial insight into the initial pattern of the relationship among variables, encouraging the researcher to examine the predictive relationships in study variables through multiple regression analyses. Moreover, moderation analysis through hierarchical regression analyses was executed to examine hypotheses testing for the intervening role of support from society and self-efficacy between the relationship of self-efficacy and stigma, and support from society and stigma, respectively. 

## 3. Results

**Figure 1:**
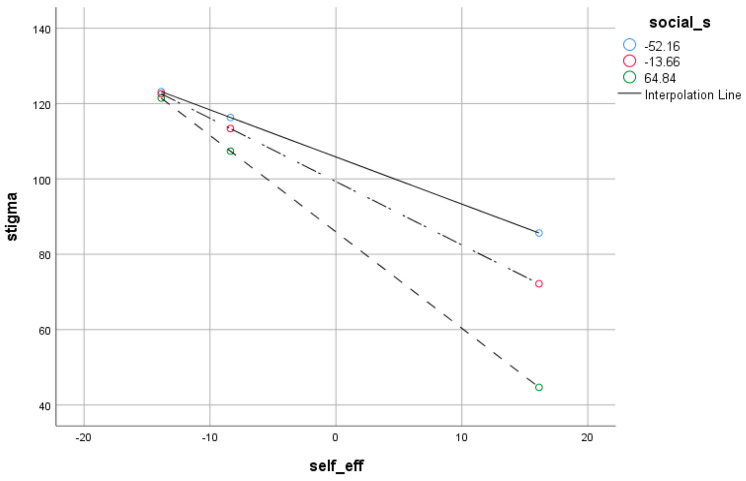
Mod Graph Showing the Interaction Effect of Social Support and Self-Efficacy.

**Figure 2:**
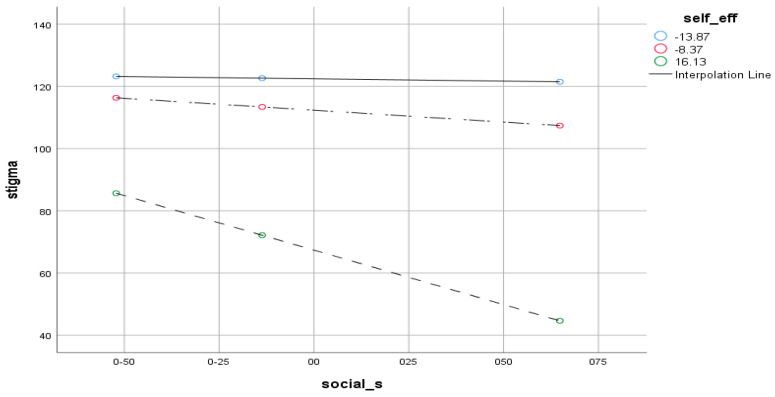
Mod Graph Showing the Interaction Effect of Self-Efficacy and Social Support.

**Figure 3:**
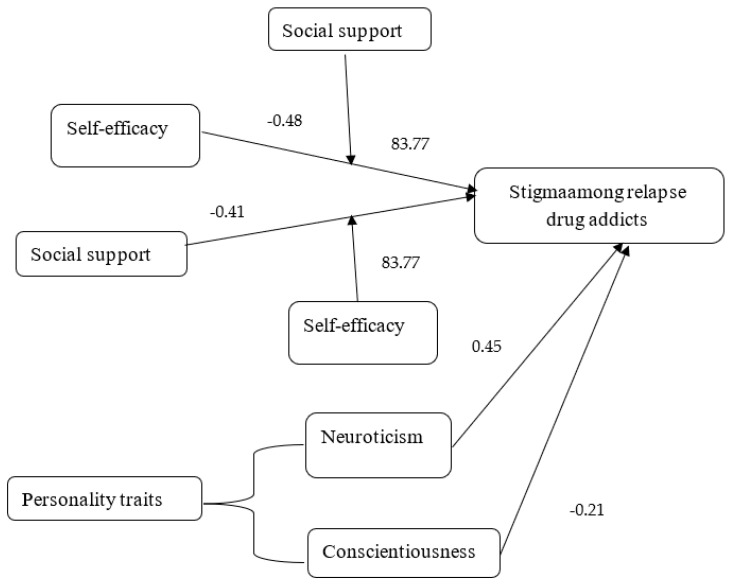
Outcome model of study findings.

Reliability analysis revealed reasonable internal consistency of self-efficacy, all the subscales of NEO-Five Factors inventory, social support, and the stigma instrument. Pearson product–moment correlation indicated a significant correlation between self-efficacy, social support, conscientiousness, neuroticism, and stigma. An analysis of regression exhibited the significant influences of self-efficacy, social support, personality traits and stigma on causing drug relapse. Analysis of moderation showed that a negative relationship between self-efficacy and stigma is stronger when social support is low. It also indicated that the negative relationship between social support and stigma is stronger when self-efficacy is low. The reliability analysis (Table 1) indicates that the reliability coefficients of all variables are above 0.70, which indicates satisfactory internal consistency. The findings (Table 2) indicated that self-efficacy has a significant positive correlation with extroversion (*r* = 0.62, *p* < 0.001), openness (*r* = 0.61, *p* < 0.001), agreeableness (*r* = 0.60, *p* < 0.001), conscientiousness (*r* = 0.32, *p* < 0.001) and social support (*r* = 0.46, *p* < 0.001). It also has a significant negative correlation with neuroticism (*r* = −0.64, *p* < 0.001) and stigma (*r* = −0.78, *p* < 0.001). The findings indicated that the predictors could account for 72% variance in stigma with *F* (4, 111) = 43.49, *p* < 0.001. The findings indicate that social support and self-efficacy have significant negative, neuroticism has significant positive, and conscientiousness has a negative but non-significant effect on stigma. The results (Table 3) demonstrate the moderating effect of social support where the whole model was revealed to be significant {ΔF (3,113) = 11.36, *p* < 0.001}. Among moderators, self-efficacy (*B = 0.16, p* < 0.001) and social support (*B = −0.17, p* < 0.001) were also found to be significant predictors of stigma. The total variance attributed to the model was 3.1% (ΔR^2^ = 0.031). Steep lines in the mod graph (Figure 1) indicate that the negative association between self-efficacy and stigma is stronger when social support is low. It is elucidated that lower social support significantly moderates the association of self-efficacy and stigma. (Table 4) demonstrated the moderating effect of self-efficacy where the whole model was revealed to be significant {ΔF (3,112) = 11.36, *p* < 0.001}. Among moderators, social support (*B = 0.17, p* < 0.001) and self-efficacy (*B = −1.83, p* < 0.05) were also found to be significant predictors of stigma. The total variance attributed to the model was 3.1% (ΔR^2^ = 0.031). Steep lines in the mod graph (Figure 2) specify that the negative association between social support and stigma is stronger when self-efficacy is low. Figure 2 demonstrated that a low level of self-efficacy significantly moderates the association between social support and stigma. Figure 3 is the outcome model of the present study that depicts the relationship pattern among variables including predictive, outcome, and moderating variables besides the inclusion of correlation coefficients and F statistics i.e., 83.77. 

Figure 1 demonstrates the moderating effect of social support. The steep line indicates that a high level of social support strengthens the negative relationship between self-efficacy and stigma.

Figure 2 depicts the moderating effect of self-efficacy. The steep line indicates that a high level of self-efficacy strengthens the negative relationship between social support and stigma.

## 4. Discussion

The current study aimed to explore psychosocial elements such as self-efficacy, social support, stigma, and personality traits in causing addiction relapse among youth in Punjab, Pakistan. The present study’s findings explored a significant influence of self-efficacy, social support, stigma and personality traits of conscientiousness and neuroticism in producing addiction relapse. The research exploration showed a negative effect of self-efficacy in causing addiction relapse. A recent study also supports our findings showing that among the factors related to relapse, the opioids group ranked significantly higher on hankering, professed denunciation, and had a low score on self-efficacy [35]. Another study also supported this finding by verifying that self-efficacy is a variable controlling the association between adolescents’ internet game addiction and aggression [36].

The study’s findings also showed a negative impact of social support in causing addiction relapse. A previous study also supports this finding by revealing that giving assistance to Alcoholics Anonymous throughout treatment dramatically reduced the risk of relapse and violent criminality [37]. There is another study that supports the above findings of our present study. This study investigated the influence of social support and self-efficacy beliefs in predicting relapse into addiction. The outcomes of this study displayed that the best indicators of relapse into addiction were self-efficacy beliefs and social support [38]. The outcomes of this analysis explored that the personality trait of conscientiousness has a significant negative impact on causing addiction relapse. A study was conducted by [39], who supported this finding by showing that conscientiousness not only improves the chances of health hazard behaviour but also influences the systems that control heroin abuse maintenance cessation. Other study results also showed that the personality trait of neuroticism has a significant positive impact on addiction relapse. A previous study also supported these findings by showing that opioid-addicted individuals rated high on neuroticism and lower on extraversion and conscientiousness [40]. Another study also supported this finding by performing a meta-analysis which exhibited that involvement with liquor was correlated with high neuroticism, lower level of conscientiousness and low agreeableness [41].

The current study has some limitations that might restrict the generalisability of its results on a larger scale. Firstly, quantitative research does not produce in-depth information like qualitative research does. So, the information gathered was not enriched and extensive. Therefore, it is suggested for future researchers that qualitative data must also be collected in addition to quantitative data. Secondly, social desirability can potentially threaten the research’s internal validity because it was a self-reported measure. Therefore, it is recommended that further studies apply a multi-method tactic rather than relying only on survey research. Thirdly, the sampling procedure applied in this research was purposive sampling. Thus, it can raise doubts concerning the representativeness of the sample.

Consequently, it is recommended that data must be gathered from respondents by applying a technique of random sampling. Finally, the sample consisted of only male addicted to drugs who have relapsed; no female participants were included in the study. Therefore, it is suggested for further studies that data should be taken from both males as well as females with substance use disorder to increase the generalisability of the findings.

All things considered; the current research suggests several practical implications. The study expands current understandings of stigma as an obstacle to healing during the conjunctive assessment of behavioural characteristics in social situations comprised of uninsured persons. The study’s results will be constructive for individuals’ perception of their self-beliefs. Furthermore, these findings will provide authentic ways for psychologists and clinical practitioners involved in drug rehab treatment to give fruitful information and raise awareness about specific problems related to an individual’s beliefs, about their capacities and potential to perform well, and how a patient’s pessimistic attitude can destroy their life by forcing them into drug abuse and relapse. The study will also provide the family members of people addicted to drugs with a greater awareness about how their support can help their family member with addiction to integrate back into everyday life, and how a lack of support from them can conversely destroy their life entirely by leading them to relapse.

## 5. Conclusions

The current study investigated the psychosocial factors responsible for causing addiction relapse. It was concluded that social support, self-efficacy, stigma and personality traits are the most critical factors responsible for causing addiction relapse. These variables also significantly correlate, as the outcomes revealed a positive association between self-efficacy and social support and a negative correlation between self-efficacy and stigma among people addicted to drugs who have relapsed. The results also proved the negative correlation between consciousness and the positive correlation of neuroticism with stigma among people addicted to drugs who have relapsed. It was also found that the negative association between self-efficacy and stigma is stronger when social support is high, and the negative relationship between social support and stigma is stronger when self-efficacy is high. The current findings encompass valuable implications in clinical and counselling settings alongside contribution to the existing literature. Future endeavours in this domain should be extended through multimethod research to overcome certain limitations.

### Limitations and Suggestions

The current study data were collected from the drug rehabilitation centres in four different cities in Pakistan, and people addicted to drugs including heroin, alcohol, and opiates, and who have relapsed were part of the study. Therefore, it is suggested that other cities and various types of drug addiction relapse should be included in future research. Additionally, maybe future research should focus on the relapse patient group for one specific drug to comprehensively understand the specific psychosocial correlates of the relapse for that particular drug.

The sampling technique used in this study was purposive convenient sampling. Therefore, it can raise doubts regarding the representativeness of the sample. It is suggested that data must be collected from participants by using a random sampling technique. In the present investigation, because of the small size of the purposive sample, the results cannot be generalised to a greater population; so, it was suggested that future research should be based on a larger sample.

The study involved quantitative research which does not produce in-depth information like qualitative research does. So, the information gathered was not enriched and extensive. It is suggested that future researchers may also benefit from utilising qualitative data in addition to quantitative data.

## Figures and Tables

**Table 1:** Descriptive Statistics, Alpha Reliability and Pearson Correlation of Variables (N = 116).

Variables	*M*	*SD*	*α*	1	2	3	4	5	6	7	8
1. Self-efficacy	23.25	11.58	0.70	-	−0.64 ***	0.62 ***	0.61 ***	0.60 ***	0.32 ***	0.46 ***	−0.78 ***
2. Neuroticism	48.25	19.06	0.96		---	−0.95 ***	−0.94 ***	−0.95 ***	−0.53 ***	−0.75 ***	0.77 ***
3. Extroversion	28.48	15.04	0.76			---	0.96 ***	0.97 ***	0.55 ***	0.76 ***	−0.75 ***
4. Openness	30.68	14.14	0.77				---	0.97 ***	0.53 ***	0.76 ***	−0.72 ***
5. Agreeableness	30.00	13.46	0.80					---	0.51 ***	0.74 ***	−0.73 ***
6. Conscientiousness	29.05	14.38	0.79						---	0.34 ***	−0.42 ***
7. Social Support	98.20	46.61	0.79							---	−0.57 ***
8. Stigma	100.48	34.71	0.75								---

*** *p* < 0.001.

**Table 2:** Multiple Regression Analysis Showing Impact of Self-Efficacy, Neuroticism, Conscientiousness and Social Support on Stigma (N = 116).

			Outcome: Stigma	
			95%CI	
Variables	Model *B*		LL	UL
(Constant)	85.77		23.17	148.38
Self-efficacy	−0.48 ***		−1.70	−0.97
Neuroticism	0.45 *		0.18	1.57
Conscientiousness	−0.21		−0.19	0.16
Social support	−0.41 *		−0.1.50	−0.92
*R* ^2^		0.72		
*F*		43.49 ***		

* *p* < 0.05. *** *p* < 0.001.

**Table 3:** Multiple Regression Analysis for the moderating role of Social Support within the relationship of Self-Efficacy and Stigma (N = 116).

		Outcome: Stigma	
Predictor	Model B		95%CI LL, UL
Constant	96.96		92.65, 101.26
Self-efficacy	0.167 ***		−2.165, −1.503
Social Support	−0.170 ***		−0.258, −0.082
Self-efficacy × Social Support	−0.003 **		−0.018, −0.005
*R* ^2^		0.692	
∆*R*^2^		0.031	
*F*		83.77 ***	
∆*F*		11.36 **	

** *p* < 0.01. *** *p* < 0.001.

**Table 4:** Multiple Regression Analysis for the moderating role of Self-Efficacy within the relationship between Social Support and Stigma (N = 116).

		Outcome: Stigma	
Predictor	Model B		95%CI LL, UL
Constant	96.96		92.65, 101.26
Social Support	−0.170 ***		−0.258, −0.082
Self-efficacy	−1.834		−2.165, −1.503
Social Support × Self-efficacy	−0.011 **		−0.018, −0.005
*R* ^2^		0.692	
*∆R* ^2^		0.031	
*F*		83.77 ***	
*∆F*		11.36 **	

** *p* < 0.001. *** *p* < 0.001.

## Data Availability

Not applicable.
